# Supplementary material for: Objective stress values during radiation emergency medicine for future human resources: Findings from a survey of nurses
Source: PLoS One. 2022 Sep 14;17(9):e0274482. doi: 10.1371/journal.pone.0274482 (PMC9473443; doi:10.1371/journal.pone.0274482)
Supplement: Attachment — Submitted filename: Objective stress values Dr Ajoke.docx [file pone.0274482.s001.docx]

Objective stress values during radiation emergency medicine for future human resources

| **S/Pages** | **Queries** | **Suggestions to the author** |
| --- | --- | --- |
| 29 & 120 | Methodology | Method  Study design should be explain clearly :-   1. If Mixed method so, justify the reason behind such study designs for this article. 2. Kindly explain the justification for the sampling strategy used in the course of this study 3. Description of tool e.g questionnaire management should be mentioned clearly   The sample size is not matching…kindly check: line 29 & 120 |
| 181 | Orderliness | Kindly rearrange by following the scientific research process hence outlining the method used clearly.  Participants characteristics in the manuscript should be properly tabulated |
|  |  |  |
