# Supplementary material for: Objective stress values during radiation emergency medicine for future human resources: Findings from a survey of nurses
Source: PLoS One. 2022 Sep 14;17(9):e0274482. doi: 10.1371/journal.pone.0274482 (PMC9473443; doi:10.1371/journal.pone.0274482)
Supplement: Attachment — Submitted filename: Response_to_Reviewers.docx [file pone.0274482.s002.docx]

Review Comments to the Author

Please use the space provided to explain your answers to the questions above. You may also include additional comments for the author, including concerns about dual publication, research ethics, or publication ethics. (Please upload your review as an attachment if it exceeds 20,000 characters)

Reviewer #1: I would like to thank authors for their interesting and timely useful study. It gain more practical knowledge as well as opportunity to intervention for keeping medical emergency treatment process intact.
Minor comment :
1.It would be better to address decontamination process in detail. This will further highlighted gaps and intervention methods.

Thank you for the advice. The following sentences were added in the Material and Methods section:

“The decontamination process in the REM seminar of FMU includes undressing patients with injury, dry decontamination, which involves wiping the body surface, and wet decontamination, which comprises wound cleaning. Similar to a previous report, the trainees performed decontamination, which prevented the spread of contamination.”

2. Authors can recommend some of the strategies to overcome stress levels during the decontamination process in the discussion section.

Stress levels during the decontamination procedure must be reduced effectively via REM seminars. However, this study did not validate the specific method used to decrease stress levels during decontamination. Nevertheless, further research must be performed to validate factors affecting stress levels during decontamination procedures. The following sentences were added in the Limitation section:

“REM includes some processes such as dressing and undressing one’s own protective gear and general patient care. However, based on the sequence of processes, the stress value was significantly high in the decontamination process. To efficiently reduce stress via REM trainings, it is necessary to decrease stress during the decontamination process. Further, education and training are essential in efficiently reducing stress during decontamination. However, specific methods for decreasing stress have not been validated.”

Reviewer #2: The authors need to state the limitations of their study and recommendations for future studies. Further, a distinct section for conclusion is recommended. Furthermore, all tables need formatting and should be assessed for readability.

Thank you for the advice. We have added separate Limitation and Conclusion sections. Moreover, we have declared that this study is observation during just a seminar, not the real radiation emergency situation as study limitation. Further, it is unclear that what kind of factor affects the stress during the decontamination process. We also have added the separate section for conclusion. Moreover, the tables were modified.

Reviewer #3: Queries Suggestions to the author
Methodology Method
Study design should be explain clearly :-
1. If Mixed method so, justify the reason behind such study designs for this article.

Thank you for the valuable comments. This was a quantitative study comparing stress values between processes via the REM seminar. The following sentence was added in the Data collection section:

“This was an observational cross-sectional study comparing differences in stress values between each of the abovementioned processes.”

2. Kindly explain the justification for the sampling strategy used in the course of this study

The following sentence was added in the Study design section:

The REM seminar of FMU was held in Fukushima City, Japan, and it included individuals who might be engaged in radiation emergency medicine in case of an actual nuclear disaster. Hence, these participants were included in the study.

3. Description of tool e.g questionnaire management should be mentioned clearly

Thank you for the advice. We have revised the sentences in the “*2.2.2. Questionnaire survey on the background characteristics of the participants”* section.

The sample size is not matching…kindly check: line 29 & 120

Thank you for pointing this out. The following sentences in the Abstract section were modified:

“In total, 74 nurses who attended the REM seminar were instructed to participate in a questionnaire (subjective) survey and to wear a shirt-type electrocardiogram (objective survey). Then, informed consent was obtained from 39 participants who were included in the analysis.”

Orderliness Kindly rearrange by following the scientific research process hence outlining the method used clearly.
Participants characteristics in the manuscript should be properly tabulated

The “2.1 Study design” section was added in the Methods section, and the Result section was rearranged (Table 1). Table 1 shows the characteristics of the participants.

Reviewer #4: The manuscript is clearly written and is scientifically sound. Recent literature published covering this topic of research include:
Carr Z, Maeda M, Oughton D, Weiss W. Non-radiological impact of a nuclear emergency: preparedness and response with the focus on health. Radiation protection dosimetry. 2018 Dec 1;182(1):112-9.
Kulka U, Wojcik A, Di Giorgio M, Wilkins R, Suto Y, Jang S, Quing-Jie L, Jiaxiang L, Ainsbury E, Woda C, Roy L. Biodosimetry and biodosimetry networks for managing radiation emergency. Radiation protection dosimetry. 2018 Dec 1;182(1):128-38.

Thank you for suggesting the abovementioned articles. Some data were added in the Discussion and Limitation sections, and their references were included.

“The current study aimed to achieve the final goal of facilitating nuclear disaster response activities. Previous reports have shown that education and training far beyond the current level can help achieve the abovementioned goal. Therefore, to efficiently conduct education and training in the future, this study first objectively evaluated actual stress status. The results are applicable globally and can be utilized in REM training using the REMPAN network worldwide.”

“To reduce anxiety/stress, it might be important to inform participants during REM training that not only dosimetry with personal dosimeters but also dosimetry with chromosomes can be performed in case of radiation exposure.”

There are a few recommendations that are suggested:
Introduction: The main aim of the study should be clearly highlighted in the last paragraph emphasising the significance of the study and what it adds to the existing knowledge base.

Thank you for the advice.

The following sentences were added in the last paragraph of the Introduction section to emphasize the goal of this study:

The current study aimed to identify the points that should be considered when maintaining sufficient human resources to achieve the final goal of facilitating nuclear disaster response activities. To attain the abovementioned goals, the negative chain of stress and anxiety (Fig 1) should be inhibited, and this study aimed to provide a solution to this problem by assessing objective stress values.

Methodology: The study design should be briefly explained and justified with a brief sub-heading

Some subheadings (e.g., 2.1–2.5) were revised.

Discussion: The limitations of the study can be highlighted more clearly and its suggested that they are re-written.

Thank you for pointing this out. We have added a separate Limitation section. Moreover, we have declared that this study is observation during just a seminar, not the real radiation emergency situation as a study limitation. Further, it is unclear the kind of factor affects the stress during the decontamination process. Nevertheless, to determine specific strategies that should be used to reduce stress effectively, further studies should be conducted to identify factors associated with elevated stress levels.

Reviewer #5: Comments to the Author
I want to congratulate the authors on this clear and well-written manuscript.
Overall, this high-quality manuscript has implications for policymakers and other concerned bodies. But there are some points of consideration for revision, but these changes are not likely to be substantial. I, therefore, recommend the following minor revisions.
INTRODUCTION
- Well-written
- author state in line with 46 “In recent years” this is not a good word because what does it mean how many years are called as recent years, so please be specific or try to put the time period.

Thank you for pointing this out. The term was changed to “After the 1990s.”

- the author needs to revise the sentence In line with “57- 63” the statement was to wordy please try to summarize it.

The following sentences were edited:

“The nuclear disaster caused by an accident at the Fukushima Daiichi Nuclear Power Plant (FDNPP) after the Great East Japan Earthquake on March 11, 2011, is one of the most disastrous events in recent years. Crisis response professionals have concerns about the risk of radiation exposure in the general population after the FDNPP accident. During the initial response, hospital staff at medical institutions receiving victims experienced strong feelings of anxiety due to the risk of radiation exposure, which affected the smooth acceptance of patients in the hospital.”

- the sentence in line with “80-84” have to written here in the introductory part, I think they are part of result of this study.

According to your advice, the following sentences were revised to make them more understandable. Moreover, the legend of Figure 1 was retained.

“Therefore, the shortage of human resources in this field is attributed to the negative chain of events caused by anxiety and stress (Fig 1). Lack of knowledge causes radiation exposure-related anxiety among workers, which reduces their intention to engage (14). This resulted in a shortage of human resources and educators, thereby making it impossible to provide sufficient education and increase the knowledge of workers (14, 20-23). Hence, this negative chain should be suppressed to smoothly provide nuclear disaster medical services.”

METHODS
- well-written
- there is a need of separate subheading which indicates study area, study design, participant selection, and data collection technique.

We have revised the subheadings according to your advice. The Methods section was also modified. That is, “study design,” “patient selection,” and “data correction” subheadings were added to clarify the contents. Our study field was medical education.

RESULT
- well-written
- How authors classified that the level of Anxiety as “Extreme, Moderate, Little, and None” this not clear, because there have to be predefined criteria or scale of measure to classify the anxiety level.

The survey used a self-administered questionnaire with a 4-point Likert scale. The “*2.2.2. Questionnaire survey on the background characteristics of the participants*” section and Table 1 and 4 footnotes were added.

DISCUSSION
- Well-written
- LIMITATION OF THE STUDY: if possible, to mention it’s recommended.

Thank you for the advice. A separate Limitation section has been added.

- CONCLUSION AND RECOMMENDATION: - it’s good if there is conclusion and recommendation headings. Also, recommendation for future direction.

Thank you for the suggestion. A separate Conclusion section has been added.

6. PLOS authors have the option to publish the peer review history of their article ([what does this mean?](https://journals.plos.org/plosone/s/editorial-and-peer-review-process#loc-peer-review-history)). If published, this will include your full peer review and any attached files.


If you choose “no”, your identity will remain anonymous but your review may still be made public.


**Do you want your identity to be public for this peer review?** For information about this choice, including consent withdrawal, please see our [Privacy Policy](https://www.plos.org/privacy-policy).

Reviewer #1: No

Reviewer #2: No

Reviewer #3: No

Reviewer #4: **Yes:** Dr Muslim Abbas Syed

Reviewer #5: **Yes:** Astawus Alemayehu
