# Supplementary material for: Objective stress values during radiation emergency medicine for future human resources: Findings from a survey of nurses
Source: PLoS One. 2022 Sep 14;17(9):e0274482. doi: 10.1371/journal.pone.0274482 (PMC9473443; doi:10.1371/journal.pone.0274482)
Supplement: Attachment — Submitted filename: Response to Reviewers.docx [file pone.0274482.s004.docx]

Title – human resource is too general. Can you be more specific which class of worker? Nurses?

Thank you for your comment. We have changed the title as follows: “Objective stress values during radiation emergency medicine for future human resources: Findings from a survey of nurses.”

Abstract – organisation OK

First para (Line 48-58, Page 46 PDF) – The introduction of the first paragraph is insignificant to this study. It is too general. Please rewrite and be more specific to the title.

Thank you for your advice. We have added the following sentences to show the significance of our study: “In particular, nurses account for approximately 40% of healthcare workers, and understanding their current status is crucial for efficiently securing human resources for the entire healthcare workforce.”

First para - all is about manpower, human resource. The author should narrow it. Nurses? Can you define in first para which class of worker? Who is going to engage in radiation emergency medicine in case of an actual nuclear disaster?

Thank you for your questions. The majority of healthcare workers are nurses, accounting for 40.2%, while physicians account for 10%. In practice, nurses also comprise the largest number of healthcare workers in radiation emergency medicine.

First para – does the nurses participated in this study have working in nuclear disaster emergency response in Fukushima? If not the the title or anything related to nuclear disaster emergency response must be stripped, and replaced with clinical procedure/diagnostic/treatment using radiation/radionuclides or just nuclear medicine practices.

Thank you for your comment. Since the nuclear disaster in Fukushima in 2011, Fukushima Medical University has accepted a large number of exposed and contaminated patients. We have responded to the nuclear disaster as an organization, and the nurses employed at our institution have earnestly acted. Thus, we believe that the title of our study is appropriate because the training given at our radiation emergency medicine seminars addresses the response to nuclear disasters.

We have changed the sentences in lines 90-93 as follows: “Since the FDNPP accident, Fukushima Medical University (FMU) has been working to accept patients exposed to or contaminated with radiation related to the FDNPP accident. Furthermore, FMU continues to develop human resources related to radiation emergency medicine (REM) and presents over 20 REM-related seminars annually.”

Line 96-98 – Redundant to a line in previous para.

Line 98-100 - Redundant to previous para

Thank you for noting these instances. We have removed the figure legends and retained only the figure title.

Line 104-105 – 20 seminar each year?

Yes, FMU conducts over 20 seminars annually. In other words, we hold seminars approximately twice a month.

Line 119-124 – redundant

We have deleted the text as per your comment.

Line 127-129 – redundant

We have deleted the text as per your comment.

Line 137 – remove “As mentioned in the Introduction”

We have deleted the text as per your comment.

Data collection 2.3, Line 158 - Use of electrocardiogram (HRV, RR) to measure average stress values. This is the main instrument used for this work. I would like you to extend the discussion, reference of other studies that support this method in your “literature work part” in introduction. I believe it will be impactful to reader as readers need to know on what hyphothesis or basis that you used to measure stress value.

Thank you for your valuable advice. We have added the following sentences to the Introduction section.

“A widely known method for objectively indicating stress levels is the evaluation of sympathetic nerve activity based on HRV (29, 30). HRV refers to the variation in the RR interval of the electrocardiogram, which indicates the instantaneous heart rate, and is affected by the balance between the sympathetic and parasympathetic nervous systems. The low-frequency/high-frequency ratio (LF/HF) obtained from the analysis of the HRV frequency is used as a surrogate parameter for sympathetic overactivity and is associated with stress load (31-33). There are several reports on the validity of HRV in assessing stress levels (34-37). Furthermore, with advances in technology, it has been suggested that wearable devices capable of measuring HRV can be used to determine stress levels (30, 38).”

Line 192 – survey on background characteristics? Please clarify the justification of the need for this survey. Why it is not a survey to assess worker understanding on radiation exposure, effects and radiation emergency situation and risk? And the question is too brief to identify or confirm the alct of knowledge or fundamental among radiation emergency workers?

The primary endpoint of this survey was comparing the stress levels for each process during the REM seminar to identify those with the highest stress load. A difficult, complex, and time-consuming questionnaire may induce stress in itself and consequently affect the stress value of the seminar process. Therefore, the questions asking about background characteristics to be answered before and after the seminar were simply designed so that they could be completed within 5 minutes.

It has been reported that interest in emergency medicine and experience in disaster activities are related to an interest in nuclear disaster medicine, and the questionnaire in our study was developed with this in mind. In addition, we asked questions about the recognition and knowledge of radiation exposure and established their own acceptable standards of radiation exposure dose. Thus, the questionnaire responses represent the participants' understanding of radiation exposure, radiation effects, and radiation emergency situations.

We have added the following sentence to the revised manuscript: “Yamada et al. (41) reported that interest in emergency medicine and experience in disaster activities are related to an interest in nuclear disaster medicine, and the questionnaire in our study was developed with this in mind.”

Methodology, Line 204 - Please extend the statistical technique used in this work? In order for the readers to understand the test and results, I believe it is useful to address the flowchart to describe what test has been used? And to test what parameter? Eg. T-test, z-test, ANOVA to check difference of background factors in two groups.

As stated in section 2.4. (Statistical analysis), we used *t* test for age and Fisher's exact test for other background factors presented in Tables 1 and 4. Stress values were compared using *t* test and Tukey–Kramer test for multiple comparisons in Table 2, Table 3, and Fig 3.

Line 256 – 260 - The highlighted text are appropriate in introduction.

Thank you for pointing this out. We have moved these sentences from the Discussion to the Introduction section.

Line 206 – 201 – remove. Please straight to the point. Discussion is to discuss your analysis/data obtained in Result

We have responded to your comment assuming that you are referring to lines 266–271, not lines 206–201.

We interpreted your comment as a reference to the lack of result data values in the Discussion section of the manuscript. Thus, we have added results to the Discussion section of the revised manuscript.

Line 288 -289 – “The present study found no difference in stress values between the group interested in nuclear disasters and that with no interest” need P-value that you have calculated to support.

Thank you for your comment. We have added p-values to the revised manuscript.

Discussion – major revision. In discussion it would be useful to state you finding and cite your analysis values. Please revise thoroughly, each statement of finding given in discussion must come along with any measurable/evidence eg. P-values, % etc.

As per your comment, we have added p-values and numbers to the Discussion section of the revised manuscript.

Conclusion – no conclusion has been drawn.

Thank you for pointing this out. Accordingly, we have added the following sentence to the Conclusion section of the revised manuscript: “The use of a shirt-type electrocardiogram made it possible to assess stress levels during the activities presented at the REM seminar. The stress load was highest during the decontamination process.”
