# Supplementary material for: Objective stress values during radiation emergency medicine for future human resources: Findings from a survey of nurses
Source: PLoS One. 2022 Sep 14;17(9):e0274482. doi: 10.1371/journal.pone.0274482 (PMC9473443; doi:10.1371/journal.pone.0274482)
Supplement: Attachment — Submitted filename: Reviewer comments PONE-D-22-11048_R1.docx [file pone.0274482.s003.docx]

Title – human resource is too general. Can you be more specific which class of worker? Nurses?

Abstract – organisation OK

First para (Line 48-58, Page 46 PDF) – The introduction of the first paragraph is insignificant to this study. It is too general. Please rewrite and be more specific to the title.

First para - all is about manpower, human resource. The author should narrow it. Nurses? Can you define in first para which class of worker? Who is going to engage in radiation emergency medicine in case of an actual nuclear disaster?

First para – does the nurses participated in this study have working in nuclear disaster emergency response in Fukushima? If not the the title or anything related to nuclear disaster emergency response must be stripped, and replaced with clinical procedure/diagnostic/treatment using radiation/radionuclides or just nuclear medicine practices.

Line 96-98 – Redundant to a line in previous para.

Line 98-100 - Redundant to previous para

Line 104-105 – 20 seminar each year?

Line 119-124 – redundant

Line 127-129 – redundant

Line 137 – remove “As mentioned in the Introduction”

Data collection 2.3, Line 158 - Use of electrocardiogram (HRV, RR) to measure average stress values. This is the main instrument used for this work. I would like you to extend the discussion, reference of other studies that support this method in your “literature work part” in introduction. I believe it will be impactful to reader as readers need to know on what hyphothesis or basis that you used to measure stress value.

Line 192 – survey on background characteristics? Please clarify the justification of the need for this survey. Why it is not a survey to assess worker understanding on radiation exposure, effects and radiation emergency situation and risk? And the question is too brief to identify or confirm the alct of knowledge or fundamental among radiation emergency workers?

Methodology, Line 204 - Please extend the statistical technique used in this work? In order for the readers to understand the test and results, I believe it is useful to address the flowchart to describe what test has been used? And to test what parameter? Eg. T-test, z-test, ANOVA to check difference of background factors in two groups.

Line 256 – 260 - The highlighted text are appropriate in introduction.

Line 206 – 201 – remove. Please straight to the point. Discussion is to discuss your analysis/data obtained in Result

Line 288 -289 – “The present study found no difference in stress values between the group interested in nuclear disasters and that with no interest” need P-value that you have calculated to support.

Discussion – major revision. In discussion it would be useful to state you finding and cite your analysis values. Please revise thoroughly, each statement of finding given in discussion must come along with any measurable/evidence eg. P-values, % etc.

Conclusion – no conclusion has been drawn.
